# Species Delimitation in the Continental Forms of the Genus *Epicrates* (Serpentes, Boidae) Integrating Phylogenetics and Environmental Niche Models

**DOI:** 10.1371/journal.pone.0022199

**Published:** 2011-09-02

**Authors:** Paula C. Rivera, Valeria Di Cola, Juan J. Martínez, Cristina N. Gardenal, Margarita Chiaraviglio

**Affiliations:** 1 Laboratorio de Biología del Comportamiento, Facultad de Ciencias Exactas, Físicas y Naturales, Universidad Nacional de Córdoba, Córdoba, Argentina; 2 Cátedra de Genética de Poblaciones y Evolución, Facultad de Ciencias Exactas, Físicas y Naturales, Universidad Nacional de Córdoba, Córdoba, Argentina; Paleontological Institute of Russian Academy of Science, United States of America

## Abstract

Until recently, the genus *Epicrates* (Boidae) presented only one continental species, *Epicrates cenchria*, distributed in Central and South America, but after a taxonomic revision using morphologic characters five species were recognized: *E. cenchria*, *E. crassus*, *E. maurus*, *E. assisi*, and *E. alvarezi*. We analyzed two independent data sets, environmental niche models and phylogeny based on molecular information, to explore species delimitation in the continental species of this genus. Our results indicated that the environmental requirements of the species are different; therefore there are not evidences of ecological interchangeability among them. There is a clear correlation between species distributions and the major biogeographic regions of Central and South America. Their overall distribution reveals that allopatry or parapatry is the general pattern. These evidences suggest that habitat isolation prevents or limits gene exchange among them. The phylogenetic reconstruction showed that the continental *Epicrates* are monophyletic, being *E. alvarezi* the sister species for the remaining two clades: *E. crassus* - *E. assisi*, and *E. maurus* - *E. cenchria*. The clade grouping the continental *Epicrates* is the sister taxon of the genus *Eunectes* and not of the Caribbean *Epicrates* clade, indicating that the genus is paraphyletic. There is a non-consistent pattern in niche evolution among continental *Epicrates*. On the contrary, a high variation and abrupt shifts in environmental variables are shown when ancestral character states were reconstructed on the sequence-based tree. The degree of genetic and ecological divergence among continental *Epicrates* and the phylogenetic analyses support the elevation to full species of *E. cenchria*, *E. crassus*, *E. maurus*, *E. assisi*, and *E. alvarezi*.

## Introduction

Accurate species delimitation is one of the most basic tasks in systematic research. However, lineages separation is a temporally extended process that may result in populations with all or some of the following features: reciprocal monophyly, reproductive isolation, ecological divergence, and morphological distinctiveness [1], [2]. Considering this, any criterion or set of rules to define species status could fail at some level depending on the properties of the entities under study, which could lead to the recognition of more or fewer species than the existing ones [3]. Besides, in organisms displaying cryptic morphological characters, fine-scaled endemism patterns, low census size (that prevents obtaining a good sample), and/or absence of reproductive barriers (that allow the existence of gene flow among different taxa) it is particularly difficult to establish species limits using only one line of evidence, like morphological or genetic data.

In the genus *Epicrates*, nine insular species distributed in the region of the Great Antilles and the Bahamas have been recognized, and until recently only one continental species, *Epicrates cenchria* [4]. Its distribution encompasses the mainland portions of Central and South America, from Nicaragua to Argentina and the continental islands of Trinidad and Tobago and Margarita [5]. According to morphology and color pattern, nine subspecies were distinguished: *E. c. cenchria*, *E. c. maurus*, *E. c. alvarezi*, *E. c. gaigei*, *E. c. crassus*, *E. c. barbouri*, *E. c. polylepis*, *E. c. hygrophilus* and *E. c. assisi* [5], [6]. Despite the particularly wide distribution range of *E. cenchria*, covering areas with very different environmental conditions and the fact that some morphotypes present an important degree of differentiation in color patterns and meristic characters [7], it was considered as a single species. However, in the last few years, the taxonomy of this species complex has been widely discussed in several studies, based only on morphological data. Some authors elevated *E. c. cenchria*, *E. c. maurus* and/or *E. c. crassus* to the rank of full species [8], [9], [10], [11], but their conclusions lacked an extensive comparative study in the group. Others preferred to maintain all the morphotypes as subspecies [5], [12], [13]. Recently, a revision of the *Epicrates cenchria* complex based on an analysis of the morphological variation in meristic, color pattern and morphometric characters proposed a new taxonomic arrangement, which comprises the species *E. cenchria* (including the nominal forms *E. c. gaigei* and *E. c. hygrophilus*), *E. crassus* (including *E. c. polylepis*), *E. maurus* (including *E. c. barbouri*), *E. assisi*, and *E. alvarezi* [14]. However, to provide a sounder support for the independence of evolutionary lineages, it has been emphasized that it is essential to use multiple lines of evidence [15]; this approach would be particularly valuable to elucidate the validity of the last taxonomic arrangement in the continental *Epicrates* group.

Ecological divergence is an important step in the process of speciation [16] and can be evaluated using environmental niche modeling (ENM) [17], [18]. This methodology allows predicting the geographic distribution of a species and provides insights into the environmental factors affecting the distribution and the geographic limits of the different lineages. Furthermore, it can be used to shed light on whether speciation or variation (such as morphologic or genetic) is associated with divergence in the ecological niche [19].

The use of molecular markers also contributes to establish species limits since they provide solid information about the phylogenetic relationships among the taxa and/or individuals. Moreover, levels of genetic divergence among closely related recognized species can serve to systematists to infer, by comparison, the status of the evolutionary independent lineages observed in groups presenting a controversial taxonomy [20].

The aim of this study was to explore species delimitation in the continental forms of the genus *Epicrates* combining two independent data sets, environmental niche models and phylogeny based on molecular information. We also compared our results with the previously obtained from morphological data, predicting that morphologically distinctive species will also be ecologically and genetically divergent. Additionally, we assessed whether the genetic variation across lineages is associated with divergence in the ecological niche.

## Results

### Ecological modeling

After the correlation tests, thirteen independent variables were selected (Appendix S1). In the models performed with Maxent, the distributions of the species were influenced by almost all the environmental variables, but with different strength (Table 1). Temperature seasonality is the only variable that makes a large contribution to almost all models, except for that of *E. maurus* (5.8%). On the other hand, annual precipitation makes a large contribution only to the *E. cenchria* model. The assessment of the Maxent models accuracy by “receiver operated characteristics” (ROC) plots indicates that the models predicted for each continental species of the genus *Epicrates* are robust, with high values of “area under the curve” (AUC) (Table 1).

**Table 1:** Topographic and bioclimatic variables ranked according to their overall model contribution for continental species of the genus *Epicrates*.

Rank	*Epicrates alvarezi*		*Epicrates crassus*		*Epicrates assisi*		*Epicrates cenchria*		*Epicrates maurus*	
1	TS	42.1	PWQ	29.2	TS	21	AP	44.7	MinTCM	21.2
2	PWQ	17.8	TS	28.5	MinTCM	18.3	TS	23.4	PDM	21
3	PCQ	15.3	PDM	12.4	MMTR	17.5	Altitude	9.8	MMTR	18.2
4	PS	11.3	Altitude	11.3	AP	12.2	MinTCM	7.9	I	14.7
5	MMTR	5	I	5.7	PWQ	10	I	4.9	PCQ	8.1
6	AMT	3.2	AMT	5.4	PDM	9.3	PCQ	3.3	PWQ	7.5
7	PDM	1.7	MinTCM	3.3	PCQ	5	PWM	2	TS	5.8
8	Altitude	1.5	PS	1.6	I	2.6	MMTR	2	AP	1.1
9	I	0.9	MaxTWM	1.3	MaxTWM	1.5	PDM	1.5	MaxTWM	0.9
10	MinTCM	0.9	PCQ	0.7	PWM	1.3	AMT	0.2	Altitude	0.7
11	MaxTWM	0.2	AP	0.3	altitude	1.1	PWQ	0.1	PS	0.7
12	PWM	0.2	MMTR	0.2	AMT	0	PS	0.1	PWM	0.3
13	AP	0	PWM	0.1	PS	0	MaxTWM	0	AMT	0
AUC	0.977		0.975		0.937		0.87		0.962	

AUC is the area under the curve in ROC plots. Bioclimatic variables are as follows: AMT, Annual mean temperature; MMTR, Mean monthly temperature range; I, Isothermality; TS, Temperature seasonality; MaxTWM, Max. temperature warmest month, MinTCM, Min. temperature coldest month, AP, Annual precipitation, PWM, Precipitation wettest month; PDM, Precipitation driest month; PS, Precipitation seasonality; PWQ, Precipitation warmest quarter; PCQ, Precipitation coldest quarter.

**Figure 1:**
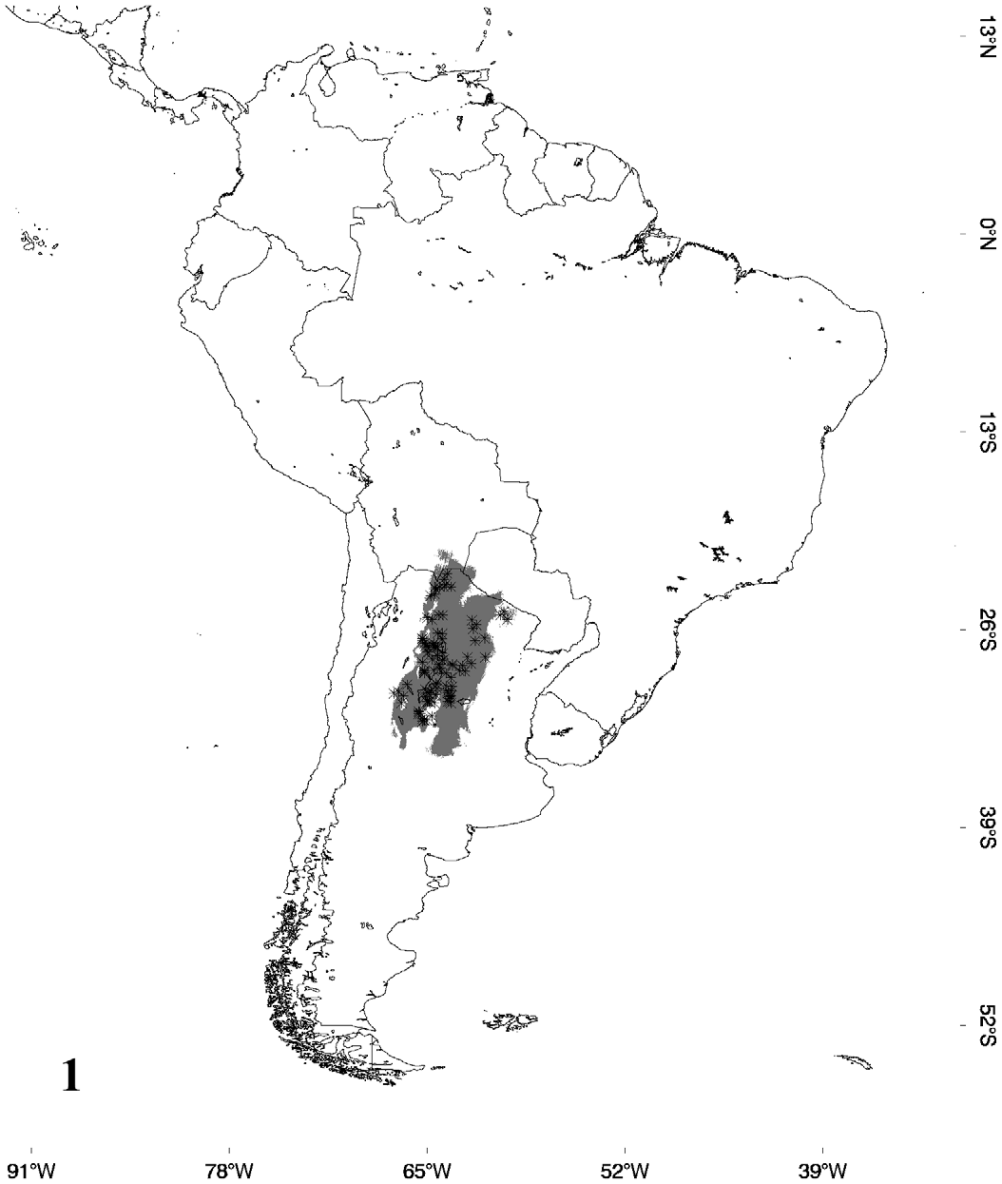
Occurrence probability map for *Epicrates alvarezi*, determined by its respective model and points of occurrence.

The predicted distribution for each species was nearly identical in Maxent and GARP. The areas of probable occurrence determined by their respective models for *E. alvarezi*, *E. crassus*, *E. assisi*, *E. cenchria* and *E. maurus* are shown in Figure 1 to [Fig pone-0022199-g002] [Fig pone-0022199-g003] [Fig pone-0022199-g004] 5.

**Figure 2 pone-0022199-g002:**
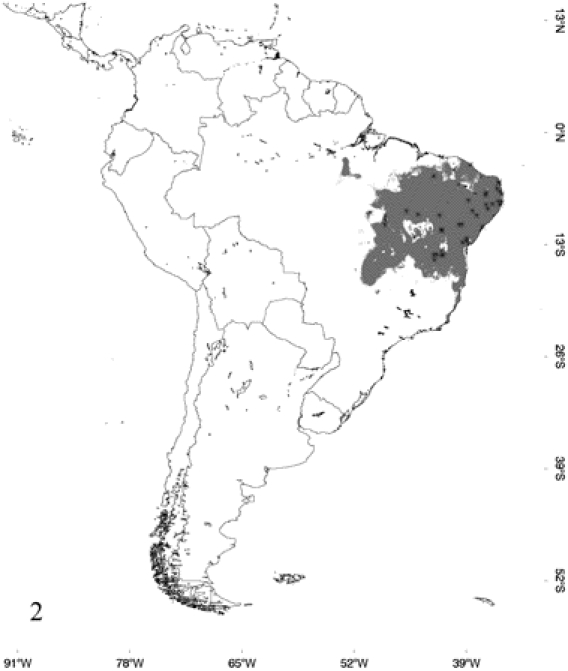
Occurrence probability map for *Epicrates crassus*, determined by its respective model and points of occurrence.

**Figure 3 pone-0022199-g003:**
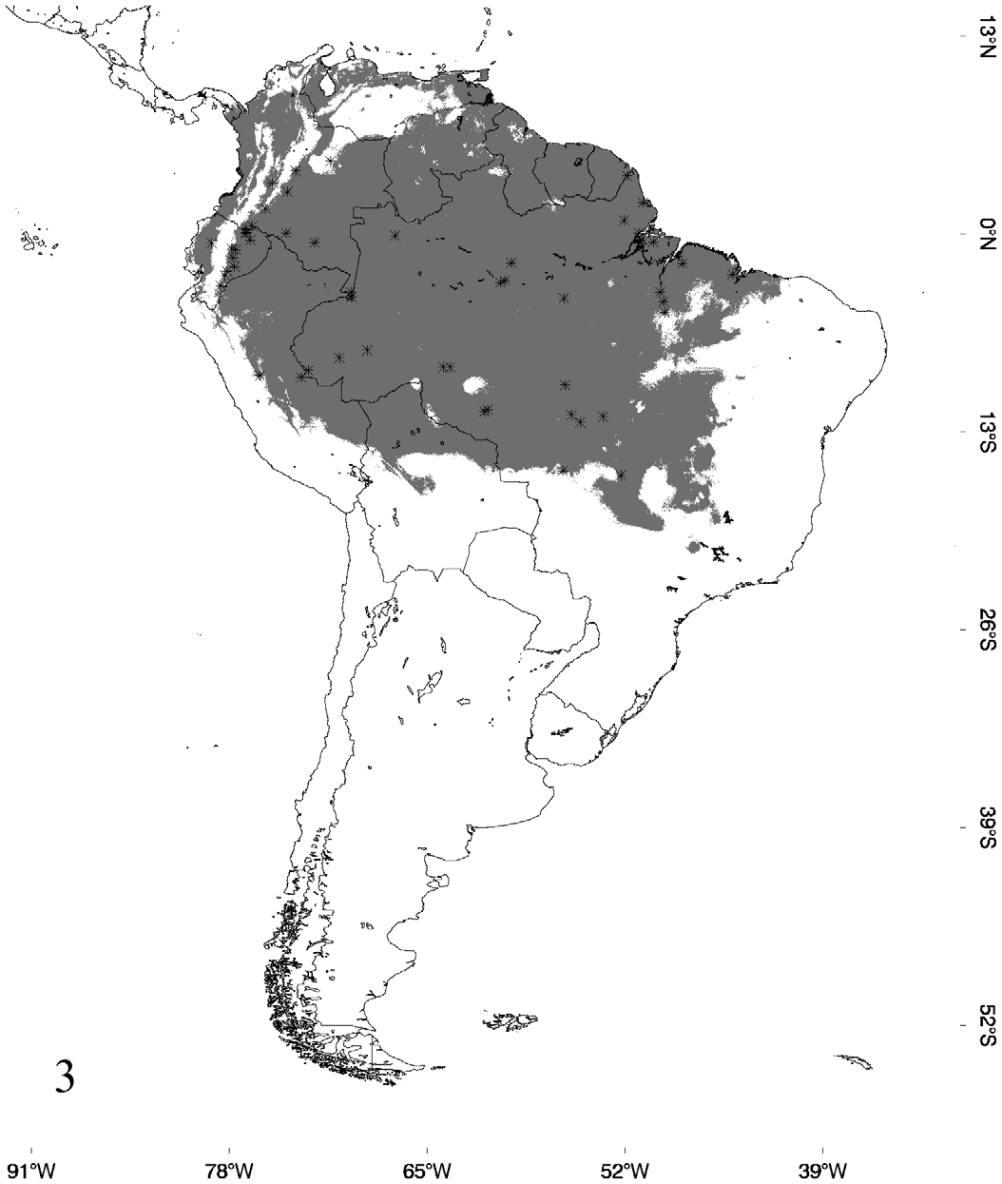
Occurrence probability map for *Epicrates assisi*, determined by its respective model and points of occurrence.

**Figure 4 pone-0022199-g004:**
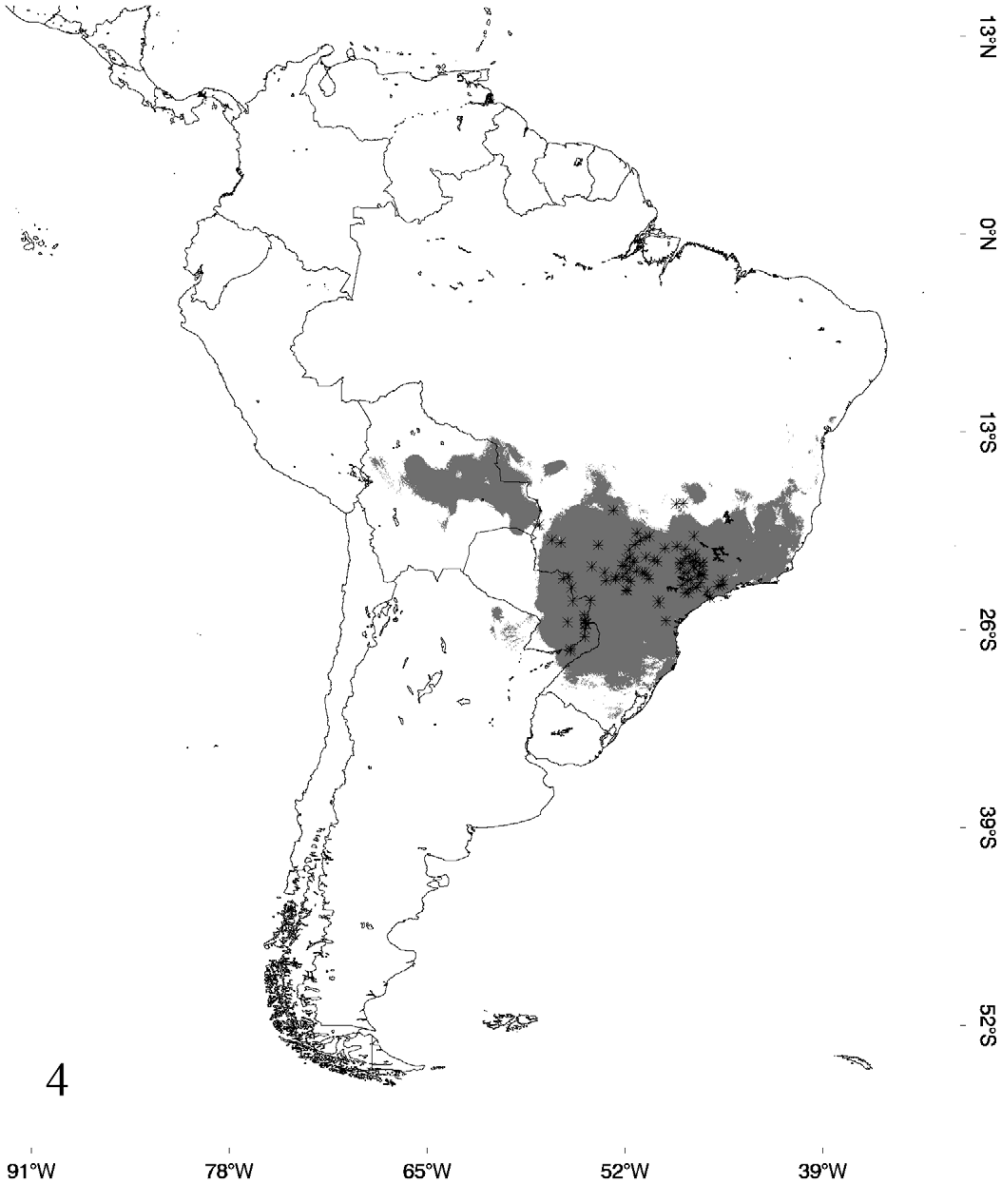
Occurrence probability map for *Epicrates cenchria*, determined by its respective model and points of occurrence.

**Figure 5:**
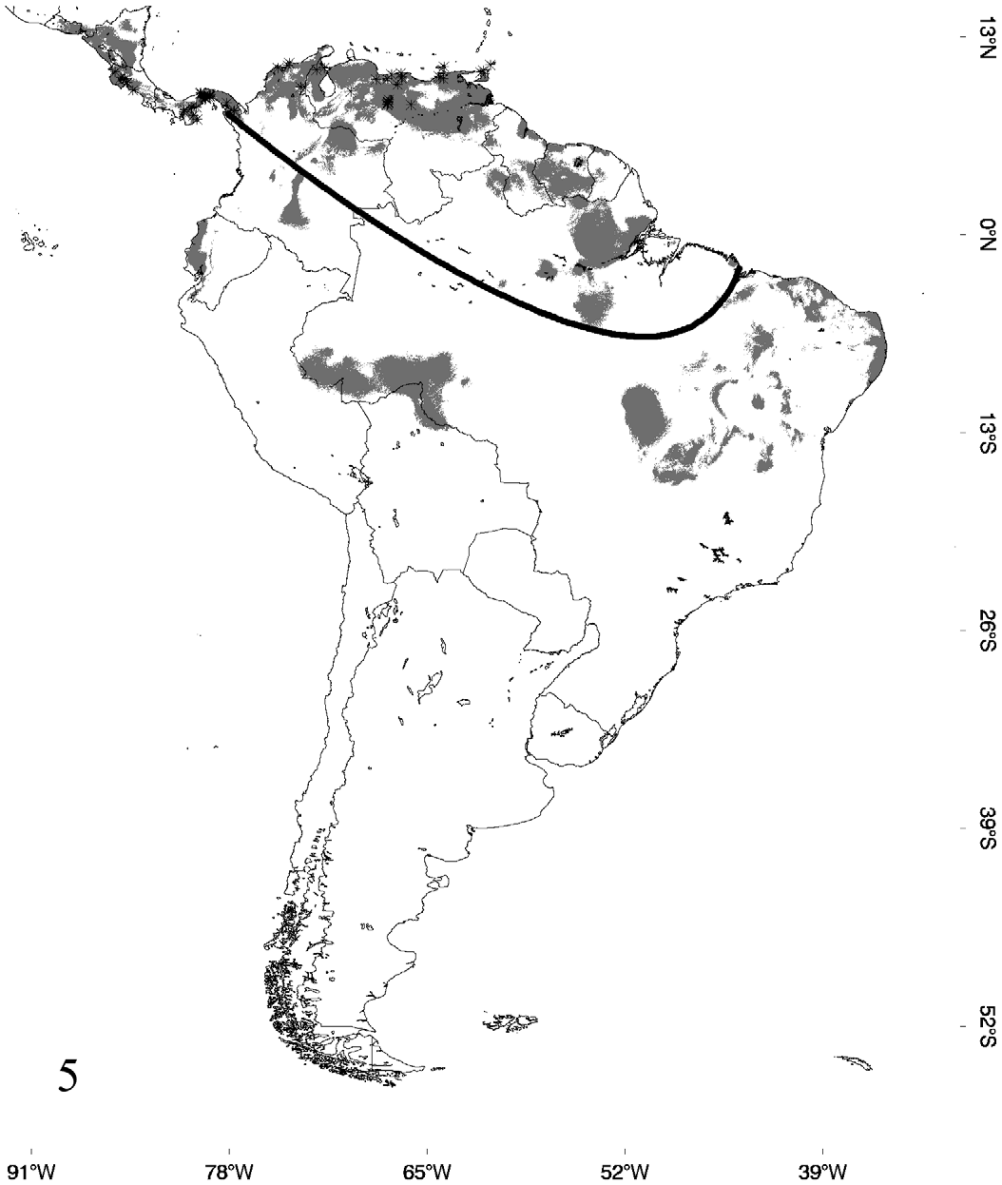
Occurrence probability map for *Epicrates maurus*, determined by its respective model and points of occurrence. The areas below the line were not considered to calculate overlapping zones between species.

According to the models, *E. alvarezi* (Fig. 1) distributes mainly in the semiarid plains and sub-mountainous areas of the Gran Chaco; it also slightly extends to the biogeographic regions of Monte, Espinal and Pampa. *Epicrates alvarezi* is the species that inhabits the southernmost area in the coldest and most arid environments of the genus distribution. In accordance with this fact, the variable that mainly contributes to the ENM of *E. alvarezi* is temperature seasonality and then, in a lesser extent, variables associated with precipitation seasonality. *Epicrates crassus* occurs in open formations of the Andean slopes, in the tropical forest of Northeast of Argentina and Central-eastern of Paraguay, in the Brazilian Cerrado and in the grassland of South and Central Brazil. The ENM indicates that the distribution of *E. crassus* is disjunct, since the model does not show suitable habitats for the species between the Eastern limit of Bolivia and Western Brazil, suggesting a barrier to gene flow between those areas (Fig. 2). Regarding *E. assisi*, the species distributes mainly in the xerophytic forest of the Caatinga region, but it also occurs in a small area of the Cerrado and in the Atlantic Rainforest of Northeast of Brazil (Fig. 3). *Epicrates cenchria* is the species most widely distributed, occupying the entire biogeographic region of Amazonas. It would be also present in the rainforest of the Pacific region, in the East of Ecuador and Colombia and in the Neotropical Savanna in North of Venezuela (Fig. 4). The ENM of *E. maurus* shows a disjunct distribution, indicating its presence in the dry forest of the biogeographic regions Pacific Coast, Venezolana, Savanna, Guajira and some areas in the Amazonic domain, in Central America and Northern South America (Fig. 5). Although this model has a high performance in the ROC plots, overpredicts the presence of the species in some extremely separated areas, as shown on the map, in Central and West of Brazil in the biographic region of Amazonas, in the East of Brazil in the Caatinga and in the rainforest of the Pacific region, in the East of Ecuador and Colombia.

**Figure 6:**
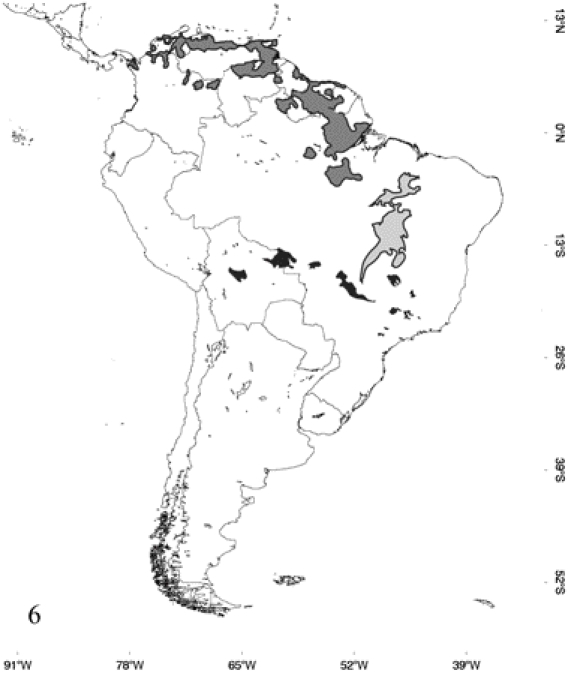
Occurrence probability maps of overlapping zones between species of continental *Epicrates*, determined by their respective models. Black areas depict overlapping between *E. cenchria* and *E. crassus*, dark gray areas between *E. cenchria* and *E. maurus*, and gray areas between *E. cenchria* and *E. assisi*.

The overall distribution of the five species reveals that allopatry or parapatry is the general pattern. Small or none overlapping zones between species were detected (Fig. 6), except for *E. maurus* that overlaps in 65% of its distribution with *E. cenchria* and *E. assisi* in 24% with *E. cenchria*. In the first case, this wide percentage of overlapping is not due to the overpredicted distribution indicated for *E. maurus* by the model, since these areas are not considered in this analysis. The map shows the sympatry between these two species in Venezuela, Guyana, Surinam, French Guiana and in the North of Brazil. The sympatry of *E. assisi* with *E. cenchria* would be occurring in the ecotone between the Caatinga and the Amazonas biogeographic regions in north western Brazil. In its entire distribution, *E. crassus* overlaps only with *E. cenchria* in a small percentage (7%), in Central and East of Bolivia and West of Brazil. *Epicrates alvarezi* distributes allopatrically with respect to all the other members of the genus *Epicrates.*

**Figure 7:**
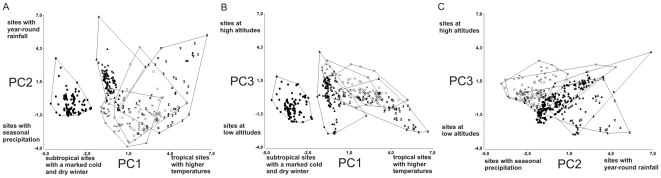
Principal components analysis (PCA) of continental species of the genus *Epicrates* for the first two PC axes (A), the first and the third (B) and the second and the third (C). The PC1 explains 51%, the PC2 explains 22% and the PC3 explains 11% of the variation. Total variation explained by the first three principal components is 84%. *E. alvarezi* (black circles), *E. crassus* (black triangles), *E. assisi* (stars), *E. cenchria* (black sandglass) and *E. maurus* (white circles).

The PCA analysis revealed three components that collectively explain more than the 84% of the variation, with each PC responsible for 51%, 22% and 11% of the variation, respectively. The PC1 depicts a gradient from subtropical sites with a marked cold and dry winter, to tropical sites with higher temperatures (Fig. 7A, 7B). The PC2 represents a gradient from sites with seasonal precipitation to localities with year-round rainfall (Fig. 7A, 7C). The PC3 mainly represents a gradient of sites at low altitudes with marked seasonality in temperature to sites at high altitudes (Fig. 7B, 7C). PCA loading scores of each variable is indicated in Appendix S1. The MANOVA analysis, using the PC scores as dependent variables and species as categorical variables, shows differences in the climatic envelopes of the five species (Wilks' lambda = 0.04, *P*≤0.001). Furthermore, the statistically significant separation among species occurs along the three PC axes (PC1 axis: *F_4_*_,360_ = 560.4, *P*≤0.001; PC2 axis: *F_4_*_,360_ = 67.3 *P*≤0.001; the PC3 axis: *F_4_*_,360_ = 38.33 *P*≤0.001).

### Phylogenetic analysis

**Figure 8:**
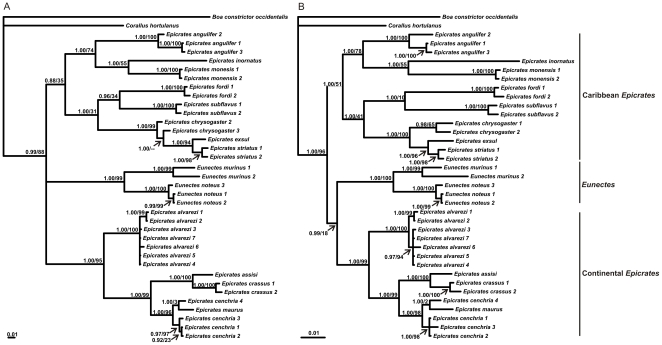
Bayesian phylogram of the 50% of majority rules consensus tree obtained from (A) the mtDNA data set (cytochrome *b* gene) and (B) the mtDNA (cytochrome *b* gene) and nDNA data set (RAG-1, NT-3 and c-*mos* genes). The topology is highly congruent to that of maximum parsimony. The node supports are as following: Bayesian posterior probabilities/bootstrap support after 1000 replicates in MP analysis.

The MP and BI phylogenetic trees of each partition produced highly congruent estimates of phylogenetic relationships among the taxa; in general, the nodes received less support in the MP analyses (Fig. 8).

The four phylogenetic trees obtained show three well supported clades (Fig. 8). The first comprises all the sequences of the Caribbean *Epicrates*, the second clade groups those of the genus *Eunectes* and the third clade clusters all the sequences of the continental *Epicrates*. Within this last clade, *E. alvarezi* is recovered as the sister taxon of the rest of the lineages of the continental *Epicrates*. *Epicrates crassus* appears as the sister taxa of *E. assisi* and *E. cenchria*, of *E. maurus*. In this last clade, one sequence of *Epicrates cenchria* (U69777; *E. cenchria* 4 in Fig. 8A) taken from GenBank groups together with *E. maurus.* Undoubtedly, this case needs a revision to confirm if the specific name of the individual from which the DNA sample was obtained is correct, according to the new taxonomic arrangement in the group. In the trees recovered using only mtDNA, the three main clades group in a polytomy. However, in the mtDNA+nDNA analyses this polytomy is resolved.

The genetic distances among the continental species of *Epicrates* were, on average, of 10.1%, ranging from 3.4% (*E. cenchria-E. maurus*) to 13.9% (*E. alvarezi-E. crassus*). *Epicrates alvarezi* is the most genetically divergent of the continental clade. The sequence U69777 presents similar levels of genetic distance with *E. cenchria* and with *E. maurus* (2.6% and 2.7% respectively); given the uncertainty about the taxonomy of the specimen, this sequence was not considered in the genetic distance analysis. Among the Caribbean *Epicrates* the values of genetic distances are the highest, 14.2% on average, ranging from 6.0% (*E. crisogaster-E. exsul*) to 17.5% (*E. angulifer-E. striatus*). Between the species of the genus *Eunectes* the genetic distance was 12.1%. Within species, the genetic distances are, on average, 0.8% and 1.4% for the continental and Caribbean *Epicrates*, respectively (Table 2–4).

**Table 2:** Kimura's two parameter (K2P) distance, for cytochrome *b*, within and among continental *Epicrates* spp.

Taxon name	1	2	3	4	5
1 *Epicrates alvarezi*	0.007				
2 *Epicrates crassus*	0.139	0.011			
3 *Epicrates assisi*	0.137	0.059	0.000		
4 *Epicrates cenchria*	0.115	0.110	0.098	0.005	
5 *Epicrates maurus*	0.103	0.105	0.110	0.034	0.000

**Table 3 pone-0022199-t003:** Kimura's two parameter (K2P) distance, for cytochrome *b*, within and among Caribbean *Epicrates* spp.

Taxon name	1	2	3	4	5	6	7	8
1 *Epicrates angulifer*	0.026							
2 *Epicrates chrysogaster*	0.163	0.020						
3 *Epicrates fordi*	0.162	0.138	0.008					
4 *Epicrates inornatus*	0.140	0.161	0.168	0.000				
5 *Epicrates exsul*	0.166	0.060	0.143	0.169	0.000			
6 *Epicrates monensis*	0.138	0.161	0.153	0.125	0.166	0.004		
7 *Epicrates subflavens*	0.162	0.133	0.116	0.155	0.147	0.143	0.011	
8 *Epicrates striatus*	0.175	0.065	0.146	0.172	0.036	0.166	0.152	0.014

**Table 4:** Kimura's two parameter (K2P) distance, for cytochrome *b*, within and among *Eunectes* spp.

Taxon name	1	2
1 *Eunectes noteus*	0.007	
2 *Eunectes murinus*	0.121	0.057

A non directional niche evolution is observed in the continental *Epicrates*, since the Mantel test correlating ecological and genetic distances between species pairs was not significant (r = 0.33, P = 0.14). Sister species pairs showed different trends: *E. maurus* presents little divergence in genetic and moderate divergence in ecological distances with *E. cenchria* (3.4 and 3.53, respectively), but it is ecologically more similar to *E. assisi* despite their genetic differences (2.71 and 11, respectively).

**Figure 9:**
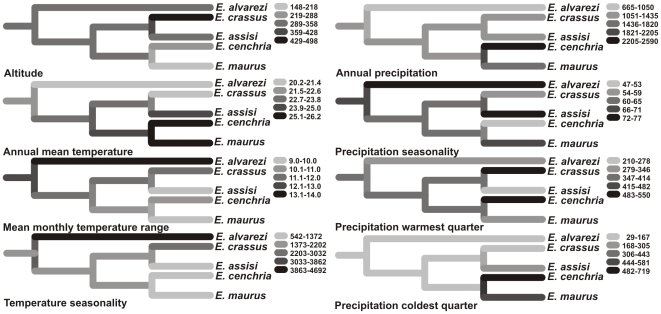
Estimated evolutionary patterns of altitude, annual mean temperature, mean monthly temperature range, temperature seasonality, annual precipitation, precipitation seasonality, and precipitation in the warmest and in the coldest quarter. Reconstruction of ancestral character states for ecological niche characters based on the phylogenetic tree derived from DNA sequences. Estimated ancestral trait values are shown at the internal nodes and visualized using a color gradient. The color gradient is described at the right of each tree.

A high variation and abrupt ecological shifts in the history of the continental *Epicrates* are shown in the reconstruction of ancestral character states of environmental variables on the sequence-based tree (Fig. 9). The reconstruction suggests that the group's common ancestor inhabited subtropical areas with intermediate elevation.

## Discussion

### Ecological divergence among the continental *Epicrates*

The environmental niche models obtained from our data are in agreement with the known distribution of each species [14], in spite of the few or even none points of occurrence in some regions (e.g. Paraguay, Bolivia and Peru). The model for *E. maurus* is an exception, since it shows several isolated areas beyond the known range of the species, located in the Amazonic forest, in the Caatinga and in the Pacific Rainforest. Nevertheless, the geographical range of an organism could be related to historical as well to ecological factors (e.g. barriers to dispersal, interspecific competition, predation pressure) [21] that are not always taken into account when modeling the potential distribution.

There are not evidences of ecological interchangeability among the continental species of *Epicrates*. This is shown by the fact that the relative contribution of each environmental parameter is taxon specific (Table 1). The PCA analysis also reveals that the environmental characteristics of the presence sites for each species are different. This result is confirmed by the MANOVA analysis, which shows that the species are well separated in the environmental space. Furthermore, there is a clear correlation between species distribution and the major biogeographic regions of Central and South America [22]. The allopatric or parapatric distribution along with the differences in the environmental requirements indicates that there is clear habitat isolation that prevents or limits gene exchange among the continental species of *Epicrates*. The wide area of simpatry between *E. maurus* and *E. cenchria*, which corresponds to the 65% of the distribution of *E. maurus*, is in accordance with previous observations in notes on the geographic distribution of these species [14], [23], [24]. Considering the absence of intrinsic reproductive barriers among species in captivity, as indicated by the commercialization of a variety of hybrid forms as pets, hybridization and gene flow between species could be occurring in the few areas of sympatry detected.

In accordance with the new taxonomic arrangement [14], the ENM of *E. crassus* encompasses the Brazilian Cerrado, where *E. c. polylepis* occurs, that of *E. maurus* includes Marajo Island where *E. c. barbouri* was described, and the model of *E. cenchria* includes the Amazonic rainforest of Peru and North of Bolivia where the presence of *E. c. gaigei* was reported. In this study was also indicated that the population of the Atlantic Rainforest in Eastern Brazil corresponds to *E. cenchria*, suggesting a disjunct distribution for the species. The specimens of this area were originally described as *E. c. higrophylus* [6], but it was synonymized with *E. cenchria* because its variation in meristic characters does not differ from the latter [14]. In our study, the ENM for *E. cenchria* does not indicate the Atlantic Rainforest of Eastern Brazil as a suitable habitat for this species, suggesting that this population is not only geographically isolated from the other populations of *E. cenchria* but it is also ecologically divergent. Furthermore, the Atlantic Rainforest was indicated as a suitable habitat for *E. crassus*. This incongruence between two sources of evidence, morphology and ENM, poses the need of new studies using another type of data (e. g. molecular) to clarify the relationships among the population of this area and the continental species of *Epicrates*.

### Phylogenetic relationships and genetic divergence among the continental *Epicrates*

This is the first molecular phylogenetic analysis of the genus *Epicrates* that includes all continental species and the majority of the Caribbean ones. The phylogenetic trees obtained with MP and BI show three well supported clades: the Caribbean *Epicrates*, the species of the genus *Eunectes* and the continental *Epicrates*. The three clades are grouped in a polytomy in the mtDNA tree, but in the nDNA+mtDNA analyses, this polytomy is resolved indicating that the Caribbean *Epicrates* are the sister taxon of the clade formed by the continental *Epicrates* and by the species of the *Eunectes* genus. This clearly indicated that the genus *Epicrates* is not monophyletic. The paraphyly of the genus was suggested in previous phylogenetic analyses of Boinae. Burbrink, using cytochrome *b* gene as molecular marker, recovered the continental *Epicrates* as the sister taxon of the Caribbean *Epicrates* and the *Eunectes* clade [25]. Noonan and Chippindale, on the base of sequences of one mitochondrial and five nuclear genes, recovered the Caribbean species of *Epicrates* outside the cluster grouping the continental *Epicrates* and *Eunectes murinus* [26]. Both studies analyzed only one sequence of the continental clade of *Epicrates*, *E. cenchria*, since they were performed before the new taxonomic arrangement [14]. Our study includes all the continental species of *Epicrates*, eight of the nine species of the Caribbean *Epicrates* and two of the three species of *Eunectes*, which greatly increases the reliability of the phylogenetic outcome obtained. Besides, the basal position of the clade grouping the insular *Epicrates* supports the hypothesis of a divergence between the South American *Epicrates* and *Eunectes*, posterior to the split of the Caribbean group.

Regarding the speciation pattern in the continental *Epicrates*, it was suggested that speciation could have been inhibited because of the high levels of gene flow that kept the populations connected [4]. Although genetic distances among the continental species of *Epicrates* are lower than those found among Caribbean species, they are similar to those obtained between the two well recognized species of the genus *Eunectes* here analyzed, thus confirming that lineage diversification has also occurred in the continental species.

The four phylogenetic trees (MP and BI) here presented clearly support the monophyly of the continental *Epicrates. Epicrates alvarezi* is recovered as the sister taxon of the clade, which contains the rest of the species. In agreement with this result, *E. alvarezi* is the most distinct taxa in morphological characters among the continental *Epicrates*, showing several exclusive features [14], and it is the species ecologically most divergent in the group. As for the other continental species, *E. crassus* appears as the sister taxon of *E. assisi* and *E. cenchria* as the sister taxon of *E. maurus*.

Within the continental *Epicrates*, there is a non-consistent pattern in niche evolution, as indicated by the lack of correlation between niche similarity and genetic distance. The reconstruction of ancestral characters on the phylogenetic tree shows important variations in environmental variables like elevation, temperature seasonality and minimum temperature of the coldest month. From a hypothetical subtropical ancestor, two different pathways diverge: on one hand, a lineage inhabiting tropical non seasonal areas, represented by *E. cenchria* and *E. maurus*; on the other hand, the branch comprising *E. alvarezi*, a species distributed in template, highly seasonal areas. However, our present data are not enough to assess a possible role of the ecological divergence in the speciation process in the group.

To sum up, the degree of genetic and ecological divergence among continental *Epicrates* and the phylogenetic analyses here performed support the elevation of *E. cenchria*, *E. crassus*, *E. maurus*, *E. assisi*, and *E. alvarezi* to the rank of species suggested by morphological data [14]. Nevertheless, the observed discrepancies between environmental niche modeling and morphology (in the population from the Atlantic Rainforest in Eastern Brazil), or between molecular phylogenetic inference and morphology (individual U69777) pose the need of new integrative analyses to elucidate discordant lineages boundaries among several sources of evidence in the continental *Epicrates*.

## Materials and Methods

### Ecological modeling

We obtained presence data from the five recognized continental species of the genus *Epicrates*. The database used includes a total of 105 points of presence of *Epicrates alvarezi*, 98 of *E. crassus*, 43 of *E. assisi*, 56 of *E. cenchria* and 69 of *E. maurus* that were gathered from the authors' field data, museum records obtained from http://splink.cria.org.br/ and http://www.herpnet.org/ and literature records [27], [28], [29], [30], [31], [32] (Appendix S2).

Two types of variables were used as predictors: topographic (altitude) derived from a digital elevation model, and bioclimatic, both obtained from WorldClim [33]. This set of environmental variables has shown to be effective data inputs for large-scale predictions of reptiles' distributions [34]. All variables were post-processed at a pixel size of 5×5 km. Resulting data layers have 714×1063 pixels and cover Central and South America between 10°N and 56°S and between 33° and 82°W. ENVI 4.5 software was used in all data analyses (http://www.ittvis.com/).

To avoid over-parameterize the analyses we extracted the environmental information from each species presence data and performed Pearson correlation tests. For pairs of variables that were highly correlated (coefficient ≥0.8), the variable considered easier to interpret biologically was chosen [18].

Several methods are used to estimate the probability area of occurrence of a species from presence-only data; these methods can result overly permissive or overly restrictive [35], which can introduce a bias in the ENMs. A method that tends to over predict ranges will probably indicate overlapped areas for parapatric lineages, leading to the spurious inference of current gene flow among them. On the other hand, an overly restrictive method will tend to indicate that the species are distributed allopatrically, showing niche differentiation where no actual ecological differences exist. To avoid this problem we used two methods, one characterized as more restrictive, the Maximum Entropy method (Maxent) [36] and the other, less restrictive, the Genetic Algorithm for Rule-Set Prediction (GARP) [37]. Maxent is used to find the probability distribution of maximum entropy subject to constraints imposed by the known distribution of the species and by the environmental conditions across the study area. All analysis were set with a convergence threshold of 1.0 E-5 with 1000 iterations; the regularization multiplier was set to 0.5, resulting in a more localized output distribution which is a closer fit to the given presence records.

GARP uses the values of environmental variables of known occurrences and pseudo-absence data to create a model of the specific requirements of the taxon. We used the “best subsets” procedure [38] based on omission and commission error statistics and the models obtained were summed up to produce predictions of potential distributions for each species. The final potential distribution models included areas where ≥5 out of 10 of the replicated models coincided in predicting presence.

A principal component analysis (PCA) was made using values of the environmental variables of each pixel with an observed presence record to examine the overall level of divergence in environmental space among the five species studied and to assess which environmental gradients separate the species. To determine whether separation in environmental space was statistically significant, a multivariate analysis of variance (MANOVA) was performed. The species were the fixed factor and PC scores were the dependent variables. A significant MANOVA score indicates potential non-overlap of ecological niche [19], [39]. In addition, each PC axis was also compared using ANOVA to assess species differentiation. All these analyses were performed using the program Infostat (http://www.infostat.com.ar/).

### Phylogenetic analysis

We studied specimens of the five continental *Epicrates*, eight of the Caribbean species of *Epicrates* and two species of the sister genus *Eunectes* (*E. notaeus* and *E. murinus*). *Corallus hortulanus* and *Boa constrictor occidentalis* were used as outgroups for phylogenetic reconstructions.

Genomic DNA was obtained from muscle tissue or scales, using a saline extraction method [40]. For phylogenetic analyses, we used the mitochondrial cytochrome *b* (Cyt-*b*) gene and three nuclear genes, neurotrophin-3 (NT-3), recombination-activating protein 1 (RAG-1) and oocyte maturation factor (c-*mos*). Primer sequences for these loci are obtained from the literature [41], [42], [43], [44] and are listed in Table 5. All sequencing reactions were performed by Macrogen USA Inc (http://www.macrogenusa.com) in an ABI PRISM 3730x1 DNA automatic analyzer (PE Applied Biosystems, Forster City, California, USA). Sequences obtained in the present study have been deposited in GenBank (Table 6). For some species, available sequences from GenBank were included in the analyses.

**Table 5:** Primer sequences, sources, aligned fragment length (FL), number of parsimony informative sites (PI) and models of sequence evolution selected for the four loci used in this study.

Locus	Primer	Primer sequence	Source	FL	PI	Selected model
Cyt-*b*	L14910H16064	GACCTGTGATMTGAAAACCAYCGTTGT CTTTGGTTTACAAGAACAATGCTTTA	[41]	1113	402	HKY+G+I
RAG-1	RAG1_f1aRAG1_r2	CAGCTGYAGCCARTACCATAAAAT CTTTCTAGCAAAATTTCCATTCAT	[42]	1053	19	GTR+G
NT-3	NT3-F3NT3-R4	ATATTTCTGGCTTTTCTCTGTGGC GCGTTTCATAAAAATATTGTTTGACCGG	[26]	664	28	HKY+G
C-*mos*	L39S78	CTGSARYTTTCTYCATCTGT CCTTGGGTGTGATTTTCTCACCT	[43], [44]	718	6	HKY

**Table 6:** List of the species and accession numbers of the sequences used in this study.

Taxon	Cytochrome *b* access	Rag-1 access	Nt-3 access	C-*mos* access
*Epicrates alvarezi* 1	HQ399506*			
*Epicrates alvarezi* 2	HQ399507*	HQ399521*	HQ399531*	HQ399541*
*Epicrates alvarezi* 3	HQ399508*			
*Epicrates alvarezi* 4	HQ399509*			
*Epicrates alvarezi* 5	HQ399510*			
*Epicrates alvarezi* 6	HQ399511*			
*Epicrates alvarezi* 7	HQ399512*	HQ399522*	HQ399532*	
*Epicrates crassus* 1	HQ399504*	HQ399520*	HQ399530*	HQ399540*
*Epicrates crassus* 2	HQ399505*			
*Epicrates cenchria* 1	HQ399500*	AY988062	AY988045	
*Epicrates cenchria* 2	HQ399501*	HQ399518*	HQ399528*	HQ399538*
*Epicrates cenchria* 3	HQ399502*			
*Epicrates cenchria* 4	U69777			
*Epicrates assisi*	HQ399503*	HQ399519*	HQ399529*	HQ399539*
*Epicrates maurus*	U69779			
*Epicrates angulifer* 1	HQ399513*	HQ399523*	HQ399533*	HQ399542*
*Epicrates angulifer* 2	U69774			
*Epicrates angulifer* 3	U69776			
*Epicrates inornatus*	U69787			
*Epicrates monensis* 1	U69792			
*Epicrates monensis* 2	U69790			
*Epicrates fordi* 1	U69784			
*Epicrates fordi* 2	U69786			
*Epicrates subflavus* 1	U69803			
*Epicrates subflavus* 2	EU138901			
*Epicrates chrysogaster* 1	U69780			
*Epicrates chrysogaster* 2	U69781			
*Epicrates exsul*	U69782			
*Epicrates striatus* 1	U69791	EU402844	EU390918	AY099966
*Epicrates striatus* 2	U69798	DQ465556	DQ465554	DQ465553
*Eunectes noteus* 1	HQ399499*	HQ399516*	HQ399526*	HQ399536*
*Eunectes noteus* 2	AM236347	AY988063	AY988046	
*Eunectes noteus* 3	U69810			
*Eunectes murinus* 1	U69809	HQ399517*	HQ399527*	HQ399537*
*Eunectes murinus* 2	U69808			
*Corallus hortulanus*	HQ399515*	HQ399525*	HQ399535*	HQ399544*
*Boa constrictor occidentalis*	HQ399514*	HQ399524*	HQ399534*	HQ399543*

*Sequences obtained in this study.

Multiple-sequence alignments were done with MUSCLE [45] using the default parameters. Phylogenetic relationships were analyzed using Maximum Parsimony (MP) and Bayesian inference (BI), for two data sets, mitochondrial DNA (Cyt-*b*) and combined mitochondrial and nuclear DNA. For MP analysis, the TNT program [46] was used considering equal weighting for all characters and gaps as missing. The Wagner algorithm was used for the heuristic search of the phylogenetic reconstructions with 250 random addition sequences, saving five trees per replica and TBR branch swapping algorithm. Then, the minimum length trees were summarized in a strict consensus tree. The nodes support was evaluated by 1000 bootstrap replicates.

For BI, the most appropriate model of sequence evolution was selected using JModeltest [47], under the Akaike information criterion. The best-fit models for each partition were implemented as partition specific models within partitioned-model analyses of the combined dataset (Table 5). Bayesian analyses were performed using MrBayes 3.1.2 [48]. The analyses were made for two million generations, with sampling intervals of 1000 generations; two independent runs were simultaneously performed on the data; each one using one cold and three heated chains. We discarded the first 25% of the samples as “burn in”. Support for tree nodes was determined according to the values of Bayesian posterior probability obtained from a majority-rule consensus tree.

The Kimura 2 parameter genetic distance (K2P) was calculated using the Cyt-*b* gene. The nuclear genes were not used to calculate genetic distances due to their low level of variability, and the absence of data for some species.

To assess whether genetic variation across species is associated with divergence in the ecological niche, a Mantel test [49] was conducted comparing matrices of genetic distance (K2P) vs. ecological distance. Ecological distances were measured as the Euclidean distance between species pairs in principal component space. Mantel tests were performed with the program TFPGA (http://www.marksgeneticsoftware.net/tfpga.htm) and 10000 permutations were used in significance testing.

To estimate overall trends in evolution of niche traits, the Phylogenetic Generalized Least Squares method (PGLS) implemented in COMPARE 4.6b (http://compare.bio.indiana.edu) was used [50]. For each species the average value of each environmental parameter extracted from the presence data were stored in the species*variables matrix. The environmental niche traits are continuous variables and we inferred their evolution with common models of continuous trait evolution. Ancestral states were calculated as the weighted average (considering within-species variation, phylogeny, and model of character evolution) of the taxa on the phylogeny.

## Supporting Information

Appendix S1 **List of selected topographical and bioclimatic variables and its PCA loading scores.**
(DOCX)

Appendix S2 **List of the presence data use for the environmental niche model in the continental** ***Epicrates.***
(DOC)
