# Supplementary material for: Species Delimitation in the Continental Forms of the Genus Epicrates (Serpentes, Boidae) Integrating Phylogenetics and Environmental Niche Models
Source: PLoS One. 2011 Sep 2;6(9):e22199. doi: 10.1371/journal.pone.0022199 (PMC3166281; doi:10.1371/journal.pone.0022199)
Supplement: Appendix S1 — List of selected topographical and bioclimatic variables and its PCA loading scores. (DOCX) [file pone.0022199.s001.docx]

**Appendix 1.** **List of selected topographical and bioclimatic variables and its PCA loading scores**

|  | PC 1 | PC 2 | PC 3 |
| --- | --- | --- | --- |
| Altitude | -0.12 | 0.41 | 0.44 |
| Annual mean temperature (AMT) | 0.30 | -0.34 | 0.01 |
| Mean monthly temperature range (MMTR) | -0.32 | 0.06 | -0.22 |
| Isothermality (I) | 0.35 | -0.03 | 0.21 |
| Temperature seasonality (TS) | -0.35 | 0.05 | -0.29 |
| Max. temperature warmest month (MaxTWM) | -0.07 | -0.45 | -0.43 |
| Min. temperature coldest month (MinTCM) | 0.35 | -0.20 | 0.13 |
| Annual precipitation (AP) | 0.34 | 0.17 | -0.17 |
| Precipitation wettest month (PWM) | 0.33 | 0.06 | 0.04 |
| Precipitation driest month (PDM) | 0.22 | 0.34 | -0.40 |
| Precipitation seasonality (PS) | -0.17 | -0.32 | 0.43 |
| Precipitation warmest quarter (PWQ) | 0.07 | 0.47 | -0.04 |
| Precipitation coldest quarter (PCQ) | 0.33 | -0.02 | -0.22 |

Values indicate the relative contributions of climatic variables to each principal component. Values ranging between [0; 1] indicate a positive contribution to the axis, while values ranging between [-1; 0] indicate a negative contribution.
