# Supplementary material for: Species Delimitation in the Continental Forms of the Genus Epicrates (Serpentes, Boidae) Integrating Phylogenetics and Environmental Niche Models
Source: PLoS One. 2011 Sep 2;6(9):e22199. doi: 10.1371/journal.pone.0022199 (PMC3166281; doi:10.1371/journal.pone.0022199)
Supplement: Appendix S2 — List of the presence data use for the environmental niche model in the continental Epicrates. (DOC) [file pone.0022199.s002.doc]

**Appendix 2. List of the presence data use for the environmental niche model in the continental *Epicrates***

| Especie | Lat* | Lon* | References | Especie | Lat* | Lon* | References |
| --- | --- | --- | --- | --- | --- | --- | --- |
| *E. alvarezi* | -22,333312 | -63,666689 | Literature record (32) | *E. alvarezi* | -22,708312 | -63,875022 | Literature record (32) |
| *E. alvarezi* | -23,041645 | -63,750023 | Literature record (32) | *E. alvarezi* | -23,208312 | -63,875022 | MACN 2422 |
| *E. alvarezi* | -23,208312 | -64,166689 | MACN 2784 | *E. alvarezi* | -23,208312 | -63,416689 | Literature record (32) |
| *E. alvarezi* | -23,249979 | -64,500022 | Literature record (32) | *E. alvarezi* | -23,374979 | -64,416689 | MACN 9876 |
| *E. alvarezi* | -23,666645 | -64,500022 | MACN 2823 | *E. alvarezi* | -23,791645 | -64,750022 | Literature record (32) |
| *E. alvarezi* | -24,791645 | -59,958357 | Literature record (27, 31) | *E. alvarezi* | -25,041645 | -60,166691 | MACN s/n |
| *E. alvarezi* | -25,041645 | -64,208356 | FML 2186 | *E. alvarezi* | -25,041645 | -63,958356 | Literature record (32) |
| *E. alvarezi* | -25,208311 | -64,958355 | MACN 2071 | *E. alvarezi* | -25,333311 | -64,666689 | Literature record (32) |
| *E. alvarezi* | -25,333311 | -59,708357 | MACN 1971 | *E. alvarezi* | -25,374978 | -62,04169 | Literature record (31) |
| *E. alvarezi* | -25,666644 | -61,750023 | Authors' field data # | *E. alvarezi* | -25,916644 | -61,958357 | Authors' field data # |
| *E. alvarezi* | -26,208311 | -64,291689 | FML 1951, FML 2255 | *E. alvarezi* | -26,249978 | -64,000022 | Literature record (32) |
| *E. alvarezi* | -26,541644 | -65,291689 | FML 244 | *E. alvarezi* | -26,541644 | -61,208357 | Literature record (32) |
| *E. alvarezi* | -26,583311 | -65,291689 | Literature record (32) | *E. alvarezi* | -26,583311 | -64,000022 | Literature record (32) |
| *E. alvarezi* | -26,708311 | -61,833357 | Literature record (32) | *E. alvarezi* | -26,708311 | -65,291689 | FML 170 |
| *E. alvarezi* | -26,874977 | -64,541689 | Literature record (32) | *E. alvarezi* | -26,874977 | -65,166689 | FML 272 |
| *E. alvarezi* | -26,999977 | -64,625022 | FML 278 | *E. alvarezi* | -27,041644 | -65,083355 | Literature record (32) |
| *E. alvarezi* | -27,124977 | -64,958355 | Literature record (32) | *E. alvarezi* | -27,124977 | -64,875022 | MACN 3080 |
| *E. alvarezi* | -27,124977 | -64,416689 | Literature record (32) | *E. alvarezi* | -27,124977 | -64,500022 | FML 246 |
| *E. alvarezi* | -27,124977 | -64,333356 | Literature record (32) | *E. alvarezi* | -27,124977 | -64,291689 | Literature record (32) |
| *E. alvarezi* | -27,45831 | -64,958355 | Literature record (32) | *E. alvarezi* | -27,45831 | -64,875022 | FML 236 |
| *E. alvarezi* | -27,541644 | -63,958356 | Literature record (32) | *E. alvarezi* | -27,666644 | -64,333356 | Literature record (32) |
| *E. alvarezi* | -27,70831 | -64,291689 | FML 1731 | *E. alvarezi* | -27,791644 | -61,16669 | Literature record (31) |
| *E. alvarezi* | -27,791644 | -62,333356 | Literature record (27, 32) | *E. alvarezi* | -27,916643 | -64,666689 | Literature record (32) |
| *E. alvarezi* | -27,916643 | -63,875022 | MACN 34376 | *E. alvarezi* | -27,999977 | -64,000022 | Literature record (32) |
| *E. alvarezi* | -28,124977 | -65,291689 | Literature record (32) | *E. alvarezi* | -28,20831 | -62,083357 | Literature record (27, 32) |
| *E. alvarezi* | -28,249977 | -64,208356 | Authors' field data # | *E. alvarezi* | -28,249977 | -63,333356 | Literature record (32) |
| *E. alvarezi* | -28,33331 | -62,66669 | Literature record (27, 32) | *E. alvarezi* | -28,33331 | -63,333356 | MACN 2041 |
| *E. alvarezi* | -28,499977 | -64,083356 | Authors' field data | *E. alvarezi* | -28,58331 | -64,166689 | Authors' field data  Literature record (32) |
| *E. alvarezi* | -28,666643 | -65,166689 | MACN 3648 | *E. alvarezi* | -28,666643 | -62,875023 | MACN 33024 |
| *E. alvarezi* | -28,666643 | -64,541689 | Literature record (32) | *E. alvarezi* | -28,70831 | -62,500023 | MACN 2468 |
| *E. alvarezi* | -28,70831 | -65,166689 | Literature record (32) | *E. alvarezi* | -28,791643 | -65,166689 | Literature record (32) |
| *E. alvarezi* | -28,83331 | -63,750023 | Authors' field data | *E. alvarezi* | -28,83331 | -65,166689 | Literature record (32) |
| *E. alvarezi* | -29,124976 | -63,416689 | Authors' field data | *E. alvarezi* | -29,499976 | -64,416689 | Authors' field data |
| *E. alvarezi* | -29,583309 | -64,333356 | Authors' field data # | *E. alvarezi* | -29,583309 | -66,291688 | Literature record (32) |
| *E. alvarezi* | -29,666643 | -63,750023 | Literature record (27) | *E. alvarezi* | -29,708309 | -63,333356 | Literature record (27) |
| *E. alvarezi* | -29,833309 | -64,333356 | Authors' field data | *E. alvarezi* | -29,916643 | -65,083355 | Authors' field data |
| *E. alvarezi* | -29,999976 | -63,625023 | Literature record (27, 29) | *E. alvarezi* | -29,999976 | -63,416689 | Literature record (27, 29) |
| *E. alvarezi* | -29,999976 | -64,750022 | Authors' field data | *E. alvarezi* | -30,041643 | -66,875021 | MACN 1234 |
| *E. alvarezi* | -30,124976 | -64,958355 | Authors' field data | *E. alvarezi* | -30,124976 | -63,625023 | Literature record (27, 29) |
| *E. alvarezi* | -30,166643 | -67,166688 | Literature record (32) | *E. alvarezi* | -30,166643 | -64,875022 | Authors' field data |
| *E. alvarezi* | -30,249976 | -64,416689 | Authors' field data | *E. alvarezi* | -30,333309 | -66,541688 | Literature record (32) |
| *E. alvarezi* | -30,333309 | -63,416689 | Literature record (29) | *E. alvarezi* | -30,374976 | -63,500023 | Literature record (29) |
| *E. alvarezi* | -30,374976 | -65,083355 | Literature record (29) | *E. alvarezi* | -30,499976 | -63,541689 | Literature record (27) |
| *E. alvarezi* | -30,708309 | -64,958355 | Authors' field data | *E. alvarezi* | -30,708309 | -64,833355 | Literature record (29) |
| *E. alvarezi* | -30,708309 | -64,875022 | Literature record (29) | *E. alvarezi* | -30,708309 | -63,625023 | Literature record (29) |
| *E. alvarezi* | -30,708309 | -63,500023 | Literature record (29) | *E. alvarezi* | -30,708309 | -65,000022 | Authors' field data |
| *E. alvarezi* | -30,791642 | -63,416689 | Authors' field data  Literature record (29) | *E. alvarezi* | -30,791642 | -64,500022 | Literature record (29) |
| *E. alvarezi* | -30,791642 | -66,666688 | Literature record (32) | *E. alvarezi* | -31,333309 | -65,541688 | Authors' field data |
| *E. alvarezi* | -31,374975 | -65,500022 | Authors' field data | *E. alvarezi* | -31,499975 | -65,416688 | Authors' field data |
| *E. alvarezi* | -31,499975 | -65,541688 | Authors' field data | *E. alvarezi* | -31.766667° | -64.633333° | Authors' field data # |
| *E. alvarezi* | -31,833309 | -65,291689 | Authors' field data | *E. alvarezi* | -31,916642 | -65,208355 | Authors' field data |
| *E. alvarezi* | -31,916642 | -65,000022 | Literature record (29) | *E. alvarezi* | -31.956234° | -65.080116° | Authors' field data # |
| *E. alvarezi* | -31,999975 | -65,291689 | Literature record (29) |  |  |  |  |
| *E. assisi* | -2,916654 | -41,791698 | IBSP 49435 | *E. assisi* | -3,749986 | -38,500033 | IBSP 20007 |
| *E. assisi* | -5,083319 | -42,791698 | IBSP 51085 | *E. assisi* | -5,208319 | -37,333366 | IBSP 51065, 52896, 53107, 53108 |
| *E. assisi* | -5,791652 | -35,208367 | IBSP 44460 | *E. assisi* | -5,833319 | -35,208367 | IBSP 45862 |
| *E. assisi* | -5,999986 | -39,833365 | IBSP 1483 | *E. assisi* | -6,499985 | -35,416701 | IBSP 20917 |
| *E. assisi* | -6,833319 | -35,125034 | MCZ R-142598 | *E. assisi* | -6,999985 | -36,708367 | IBSP 55463 |
| *E. assisi* | -7,124985 | -34,875034 | IBSP 53724, 54710 | *E. assisi* | -7,291652 | -36,750033 | IBSP 33409 |
| *E. assisi* | -7,874985 | -38,750033 | IBSP 32914 | *E. assisi* | -7,999985 | -35,291701 | IBSP 46973 |
| *E. assisi* | -8,041651 | -34,875034 | IBSP 46547 | *E. assisi* | -8,291651 | -35,958367 | IBSP 51954 |
| *E. assisi* | -8,374985 | -36,708367 | IBSP 42896, 42933, 46613 | *E. assisi* | -8,416651 | -35,083367 | IBSP 48811 |
| *E. assisi* | -8,874984 | -36,500033 | IBSP 51777 | *E. assisi* | -9,083318 | -38,291699 | MHN-UNICAMP 638 |
| *E. assisi* | -9,124984 | -45,916696 | IBSP 1690, 1691 | *E. assisi* | -9,291651 | -50,083361 | IBSP 29241 |
| *E. assisi* | -9,499984 | -44,583364 | IBSP 67523 | *E. assisi* | -9,624984 | -37,7917 | IBSP 54254 |
| *E. assisi* | -9,624984 | -42,083365 | IBSP 28706 | *E. assisi* | -9,749984 | -48,375029 | IBSP 64947, 65025, 65026, 65027, 65028, 65029, 65030, 65031, 65032, 65033 |
| *E. assisi* | -10,166651 | -48,333362 | IBSP 65240, 65241, 65335, 65337, 65340, 65451, 65518, 65519, 65520, 65648, 65649, 65650, 65651, 65652, 65653, 65654, 65655, 65656, 65657, 65658, 65659, 65660, 65789, 66304 | *E. assisi* | -10,66665 | -39,500032 | IBSP 62953 |
| *E. assisi* | -10,708317 | -39,833365 | MCZ R-142597 | *E. assisi* | -10,708317 | -48,416695 | IBSP 65426 |
| *E. assisi* | -10,91665 | -37,083366 | IBSP 49850, 53186 | *E. assisi* | -11,29165 | -41,875031 | IBSP 43890 |
| *E. assisi* | -11,374983 | -41,833365 | IBSP 55304, 55305 | *E. assisi* | -12,16665 | -45,00003 | IBSP 48154 |
| *E. assisi* | -12,29165 | -38,958366 | IBSP 20914 | *E. assisi* | -12,583316 | -39,000032 | IBSP 49309, 49310, 49333, 49334, 49335, 51119, 52257 |
| *E. assisi* | -12,749983 | -39,416699 | IBSP 25063 | *E. assisi* | -13,666649 | -41,833365 | IBSP 29817, 29818, 29819 |
| *E. assisi* | -14,166649 | -42,416698 | IBSP 3210 | *E. assisi* | -14,208316 | -41,666698 | IBSP 48585, 41087 |
| *E. assisi* | -14,208316 | -42,791698 | IBSP 50341, 53857 | *E. assisi* | -14,249982 | -41,875031 | IBSP 34456, 40374, 40379, 40384, 40542, 41145, 44052, 45721 |
| *E. assisi* | -14,791649 | -42,666698 | IBSP 25883, 25884 |  |  |  |  |
| *E. cenchria* | 4,791677 | -71,333353 | FPR 59277 | *E. cenchria* | 4,166677 | -73,625019 | FPR 59260, 59267, 59268, 59269 |
| *E. cenchria* | 3,833344 | -51,833361 | IBSP 14035, 14624, 14626, 15389 | *E. cenchria* | 3,333344 | -75,166685 | BNHM 71520 |
| *E. cenchria* | 2,750011 | -74,166685 | FPR 59278 | *E. cenchria* | 2,041678 | -50,791694 | IBSP 34104 |
| *E. cenchria* | 1,625011 | -75,625018 | FPR 59259 | *E. cenchria* | 0,875012 | -52,041694 | IBSP 19126, 24770, 25425 |
| *E. cenchria* | 0,500012 | -76,500017 | FPR 59270, 59279 | *E. cenchria* | 0,333345 | -76,875017 | FPR 59261 |
| *E. cenchria* | 0,083345 | -76,875017 | FPR 59262  KU 148285 | *E. cenchria* | 0,041679 | -51,083361 | IBSP 24828, 24829 |
| *E. cenchria* | 0,041679 | -76,958351 | FPR 59257, 59263 | *E. cenchria* | 0,041679 | -74,250018 | FPR 59265 |
| *E. cenchria* | -0,124988 | -67,083354 | IBSP 33386 | *E. cenchria* | -0,416654 | -76,625017 | KU 98608, 98609, 98610  LACM 75171 |
| *E. cenchria* | -0,541654 | -50,125028 | IBSP 23942 | *E. cenchria* | -0,583321 | -72,375019 | FPR 59275 |
| *E. cenchria* | -0,624988 | -79,166683 | CMNH 43826 | *E. cenchria* | -0,999988 | -77,83335 | CMNH 60300 |
| *E. cenchria* | -1,041654 | -77,58335 | MCZ R-173893, R-173894, R-173895, R-173896 | *E. cenchria* | -1,416654 | -48,458362 | KU 127255, 124588 |
| *E. cenchria* | -1,458321 | -48,500029 | IBSP 15028 | *E. cenchria* | -1,499987 | -48,625029 | KU 128091 |
| *E. cenchria* | -1,583321 | -77,750017 | NMNH-USNM 204100 | *E. cenchria* | -1,916654 | -59,458358 | IBSP 51873 |
| *E. cenchria* | -1,958321 | -48,208362 | IBSP 14687, 14690, 14691, 14692, 14694 | *E. cenchria* | -2,166654 | -77,666684 | NMNH-USNM 204095 |
| *E. cenchria* | -2,291654 | -78,125017 | NMNH-USNM 65485 | *E. cenchria* | -2,45832 | -78,166683 | MCZ R-145353  NMNH-USNM 283941 |
| *E. cenchria* | -2,666654 | -44,916697 | IBSP 21778 | *E. cenchria* | -2,749987 | -78,291683 | NMNH-USNM 204094, 204097 |
| *E. cenchria* | -3,08332 | -59,875024 | MCZ R-3289 | *E. cenchria* | -3,20832 | -60,166691 | MHN-UNICAMP 2038 |
| *E. cenchria* | -3,416653 | -78,58335 | KU 147193 | *E. cenchria* | -3,83332 | -49,666695 | IBSP 46410 |
| *E. cenchria* | -3,83332 | -70,00002 | FPR 59276 | *E. cenchria* | -4,166653 | -69,958353 | FPR 59264, 59266, 59271, 59272, 59273, 59274  LACM 101302  MCZ R-48966 |
| *E. cenchria* | -4,249986 | -56,000026 | IBSP 46479, 46591 | *E. cenchria* | -4,45832 | -49,458362 | IBSP 46411 |
| *E. cenchria* | -5,166653 | -49,375028 | IBSP 32820 | *E. cenchria* | -7,624985 | -68,916687 | IBSP 24140 |
| *E. cenchria* | -8,124985 | -70,75002 | MHN-UNICAMP 460 | *E. cenchria* | -8.383333 | -74.550000 | Fiel trip # |
| *E. cenchria* | -8,749984 | -63,458356 | IBSP 53122 | *E. cenchria* | -8,958318 | -72,791686 | MHN-UNICAMP 1586 |
| *E. cenchria* | -9,291651 | -76,000018 | NMNH-USNM 193694, 193695, 193696 | *E. cenchria* | -9,416651 | -73,250019 | IBSP 14642 |
| *E. cenchria* | -9,916651 | -55,916692 | IBSP 41463, 41474,46559, 48155, 51093 | *E. cenchria* | -11,54165 | -61,000024 | MZUEL 561 |
| *E. cenchria* | -11,66665 | -61,208357 | IBSP 41031, 41037 | *E. cenchria* | -11,874983 | -55,500026 | IBSP 40688, 51962 |
| *E. cenchria* | -11,999983 | -53,416693 | IBSP 46592 | *E. cenchria* | -12,374983 | -54,916693 | IBSP 55245, 55246 |
| *E. cenchria* | -15,583315 | -56,083359 | IBSP 47675 | *E. cenchria* | -15,874982 | -52,250027 | IBSP 44274, 48156, 51091, 51358 |
| *E. crassus* | -17,708314 | -48,166695 | IBSP 54996 | *E. crassus* | -17,749981 | -48,625029 | IBSP 40615 |
| *E. crassus* | -18,166647 | -52,750027 | MHN-UNICAMP 1958 | *E. crassus* | -19,12498 | -57,625025 | CMNH R320 |
| *E. crassus* | -19,666647 | -51,208361 | IBSP 46350 | *E. crassus* | -19,87498 | -47,458362 | IBSP 707 |
| *E. crassus* | -19,87498 | -50,375028 | IBSP 41805, 41811, 41821, 41826, 41843, 41910, 41916, 42047, 43046, 46964 | *E. crassus* | -19,958313 | -50,625028 | IBSP 23135 |
| *E. crassus* | -20,12498 | -56,791692 | IBSP 23721 | *E. crassus* | -20,166647 | -51,000028 | IBSP 33855, 37861, 37862, 37867, 38012, 38844 |
| *E. crassus* | -20,291647 | -56,208359 | IBSP 14360 | *E. crassus* | -20,416646 | -51,333361 | IBSP 35747, 36209,36536, 36585, 36717,36718, 36719, 37859, 37860, 37863, 37864, 37865, 37866, 37868, 38091, 38124, 38126, 38129, 38171, 38326, 38327, 38328, 38329, 38333, 38501, 38502, 38503, 38805, 38806, 38807, 38808, 38809 |
| *E. crassus* | -20,458313 | -53,750027 | IBSP 27685 | *E. crassus* | -20,541646 | -48,583362 | IBSP 21919 |
| *E. crassus* | -20,666646 | -49,375028 | IBSP 57348 | *E. crassus* | -20,708313 | -48,041695 | IBSP 49848 |
| *E. crassus* | -20,74998 | -51,708361 | IBSP 9625, 19493, 19494, 46972 | *E. crassus* | -21,041646 | -47,375029 | IBSP 15679, 22489 |
| *E. crassus* | -21,166646 | -47,791696 | IBSP 49783, 52174, 53573, 62351 | *E. crassus* | -21,24998 | -50,625028 | IBSP 28070 |
| *E. crassus* | -21,24998 | -51,666694 | IBSP 53288 | *E. crassus* | -21,333313 | -48,000029 | IBSP 698 |
| *E. crassus* | -21,374979 | -51,875027 | IBSP 22478, 22479 | *E. crassus* | -21,416646 | -50,083361 | IBSP 10978, 10839, 15712, 21917 |
| *E. crassus* | -21,458313 | -47,375029 | IBSP 53266 | *E. crassus* | -21,499979 | -51,458361 | IBSP 53656, 53681 |
| *E. crassus* | -21,499979 | -47,541696 | NMNH-USNM 165529 | *E. crassus* | -21,541646 | -49,875028 | IBSP 49060  NMNH-USNM 100726 |
| *E. crassus* | -21,541646 | -47,708362 | IBSP 53675 | *E. crassus* | -21,583313 | -51,375027 | IBSP 22783 |
| *E. crassus* | -21,583313 | -46,875029 | IBSP 16720, 53569 | *E. crassus* | -21,583313 | -48,375029 | IBSP 53676 |
| *E. crassus* | -21,624979 | -47,333362 | IBSP 10994 | *E. crassus* | -21,666646 | -48,250029 | IBSP 10844 |
| *E. crassus* | -21,749979 | -47,083363 | IBSP 1811  NMNH-USNM 165528 | *E. crassus* | -21,791646 | -52,166694 | IBSP 15538 |
| *E. crassus* | -21,833313 | -46,875029 | IBSP 15197 | *E. crassus* | -21,833313 | -52,000027 | IBSP 7095 |
| *E. crassus* | -21,874979 | -54,166693 | IBSP 54200 | *E. crassus* | -21,874979 | -47,500029 | IBSP 15373, 15688 |
| *E. crassus* | -21,999979 | -51,666694 | IBSP 19731, 45720 | *E. crassus* | -21,999979 | -48,375029 | IBSP 56923 |
| *E. crassus* | -21,999979 | -47,416696 | IBSP 29280 | *E. crassus* | -22,166646 | -51,250028 | IBSP 11022, 11023, 10919 |
| *E. crassus* | -22,249979 | -50,708361 | IBSP 24715, 24716, 24717, 24718, 24719, 24720, 24721, 24722, 24723, 24724, 24725, 24726, 24727, 24728 | *E. crassus* | -22,249979 | -53,33336 | IBSP 20815 |
| *E. crassus* | -22,249979 | -47,833362 | IBSP 12813, 51889 MHN-UNICAMP2795, 2796 | *E. crassus* | -22,249979 | -50,875028 | IBSP 20735, 21506, 27684 NMNH-USNM 165527 |
| *E. crassus* | -22,291646 | -48,125029 | IBSP 51994 | *E. crassus* | -22,291646 | -51,916694 | IBSP 52809 |
| *E. crassus* | -22,291646 | -47,125029 | NMNH-USNM 39053 | *E. crassus* | -22,374979 | -46,958363 | MHN-UNICAMP 135 |
| *E. crassus* | -22,416646 | -46,833363 | IBSP 27386 | *E. crassus* | -22,416646 | -46,958363 | IBSP 27205 |
| *E. crassus* | -22,416646 | -50,583361 | IBSP 15754, 23153, 30840 | *E. crassus* | -22,499979 | -47,208362 | IBSP 1505 |
| *E. crassus* | -22,541646 | -55,708359 | IBSP 25603 | *E. crassus* | -22,541646 | -52,166694 | IBSP 50274 |
| *E. crassus* | -22,541646 | -52,58336 | IBSP 40411 | *E. crassus* | -22,666646 | -50,416695 | IBSP 47342, 47343, 47344, 47345, 47346, 47347, 47348, 47349, 47350, 47351, 47352 |
| *E. crassus* | -22,666646 | -56,041692 | Literature record (28) | *E. crassus* | -22,708312 | -52,625027 | IBSP 19085 |
| *E. crassus* | -22,708312 | -47,666696 | IBSP 14163 | *E. crassus* | -22,708312 | -51,791694 | IBSP 46423, 50893 |
| *E. crassus* | -22,749979 | -45,583363 | IBSP 19698 | *E. crassus* | -22,791646 | -47,875029 | IBSP 2139, 2683 |
| *E. crassus* | -22,791646 | -53,250027 | IBSP 31141 | *E. crassus* | -22,833312 | -51,625027 | MZUEL 76 |
| *E. crassus* | -22,916646 | -47,041696 | IBSP 10874 | *E. crassus* | -23,041645 | -45,541696 | IBSP 59967, 55376, 55787 |
| *E. crassus* | -23,083312 | -48,250029 | IBSP 51837 | *E. crassus* | -23,083312 | -45,708363 | IBSP 10888, 63320 |
| *E. crassus* | -23,166645 | -45,87503 | IBSP 22009, 22927, 22930, 22990, 23051, 23052, 23749, 28904, 29242, 32448, 33933, 52167, 53719, 53720, 56930, 32879 | *E. crassus* | -23,208312 | -47,291696 | IBSP 20435 |
| *E. crassus* | -23,249979 | -47,291696 | IBSP 50046, 50058, 50059, 51906, 52090, 52555, 57144 | *E. crassus* | -23,249979 | -55,500026 | IBSP 40267 |
| *E. crassus* | -23,333312 | -47,708362 | IBSP 33226 | *E. crassus* | -23,416645 | -51,916694 | IBSP 21695, 32552 |
| *E. crassus* | -23,458312 | -51,833361 | IBSP 53394 | *E. crassus* | -23,499979 | -46,583363 | MCZ R-20701, R-20702, R-20703, R-20704, R-16674 |
| *E. crassus* | -23,583312 | -48,041695 | IBSP 50248 | *E. crassus* | -23,624979 | -47,833362 | IBSP 56821 |
| *E. crassus* | -23,874978 | -46,416696 | IBSP 13670 | *E. crassus* | -24,083312 | -54,250026 | IBSP 32683 |
| *E. crassus* | -24,124978 | -55,416692 | Literature record (28) | *E. crassus* | -24,124978 | -49,833361 | IBSP 40269 |
| *E. crassus* | -24,249978 | -49,708361 | IBSP 46965 | *E. crassus* | -25,041645 | -54,666693 | Literature record (28) |
| *E. crassus* | -25,416644 | -54,625026 | Literature record (28) | *E. crassus* | -25,416644 | -49,291695 | IBSP 37427, 52293, 52369, 52582, 52583 |
| *E. crassus* | -25,499978 | -55,750026 | IBSP 29113 | *E. crassus* | -25,541644 | -54,58336 | Literature record (28) |
| *E. crassus* | -25,583311 | -54,541693 | Literature record (30) # | *E. crassus* | -25,624978 | -54,541693 | Literature record (30) |
| *E. crassus* | -25,916644 | -54,625026 | Literature record (30) | *E. crassus* | -26,458311 | -54,625026 | Literature record (30) |
| *E. crassus* | -27,249977 | -55,541692 | Literature record (30) | *E. crassus* | -27,374977 | -55,583359 | Literature record (30) |
| *E. maurus* | 11,291674 | -74,041685 | CMNH R2025 | *E. maurus* | 11,166674 | -60,833357 | MCZ R-13195, R-13196 |
| *E. maurus* | 11,125008 | -74,125018 | CMNH R136 | *E. maurus* | 11,041674 | -63,875022 | NMNH-USNM 217167 |
| *E. maurus* | 10,958341 | -74,791685 | NMNH-USNM 115612 | *E. maurus* | 10,958341 | -71,750019 | MCZ R-139559 |
| *E. maurus* | 10,833341 | -72,250019 | NMNH-USNM 217168 | *E. maurus* | 10,791674 | -61,29169 | CAS 231783 |
| *E. maurus* | 10,708341 | -85,500014 | LACM 150314 | *E. maurus* | 10,666674 | -61,458357 | MCZ R-80992 |
| *E. maurus* | 10,625008 | -66,708355 | IBSP 18406, 18407 | *E. maurus* | 10,583341 | -61,208357 | TCWC 78262 |
| *E. maurus* | 10,541674 | -67,333354 | CMNH S7302 | *E. maurus* | 10,416674 | -64,208356 | KU 117033 |
| *E. maurus* | 10,375008 | -66,625021 | CMNH S7438 | *E. maurus* | 10,333341 | -85,125014 | BNHM 170796 |
| *E. maurus* | 10,333341 | -64,125022 | KU 117034 | *E. maurus* | 10,291675 | -66,666688 | CMNH S7461 |
| *E. maurus* | 10,250008 | -63,916689 | CMNH S7941  NMNH-USNM 164369 | *E. maurus* | 10,250008 | -84,833347 | LACM 150310 |
| *E. maurus* | 10,250008 | -67,583354 | IBSP 25725 | *E. maurus* | 10,208341 | -85,000014 | CMNH 107382 |
| *E. maurus* | 10,208341 | -68,333354 | NMNH-USNM 217169 | *E. maurus* | 10,000008 | -84,750014 | CMNH 107364 |
| *E. maurus* | 10,000008 | -84,708347 | LACM 150313 | *E. maurus* | 9,875008 | -66,916688 | TCWC 47260 |
| *E. maurus* | 9,708341 | -73,291685 | CAS 116291 | *E. maurus* | 9,666675 | -73,000019 | NMNH-USNM 61224 |
| *E. maurus* | 9,500008 | -84,333348 | LACM 150309, 150311, 150312 | *E. maurus* | 9,416675 | -70,00002 | KU 167566 |
| *E. maurus* | 9,333342 | -79,875016 | MCZ R-18932 | *E. maurus* | 9,291675 | -79,916683 | KU 107642  MCZ R-20570 |
| *E. maurus* | 9,250008 | -79,833349 | MCZ R-23851 | *E. maurus* | 9,208342 | -79,625016 | MCZ R-39745 |
| *E. maurus* | 9,208342 | -79,250016 | MCZ R-23846 | *E. maurus* | 9,125008 | -79,708349 | MCZ R-23848, R-23849, R-23850 |
| *E. maurus* | 9,125008 | -79,291683 | KU 110266 | *E. maurus* | 9,083342 | -79,666683 | MCZ R-23857, R-23858, R-23859 |
| *E. maurus* | 9,083342 | -79,541683 | UMNH 5682 | *E. maurus* | 9,083342 | -79,416683 | KU 110267, 110268 |
| *E. maurus* | 9,083342 | -79,291683 | UMNH 5678 | *E. maurus* | 9,041675 | -79,625016 | MCZ R-23852, R-23853 |
| *E. maurus* | 9,000008 | -79,583349 | KU 110261, 110265  MCZ R-23855, R-23856, R-23860, R-177157, R-177158, R-177159 | *E. maurus* | 9,000008 | -79,541683 | KU 80252, 107641 |
| *E. maurus* | 8,958342 | -79,583349 | UMNH 5679, 5680 | *E. maurus* | 8,958342 | -79,541683 | KU 110262, 110263  LACM116135  MCZ R-23843, R-23844, R-23845, R-23847, R-25133  UMNH 5681 |
| *E. maurus* | 8,875008 | -67,500021 | TCWC 47884, 47885 | *E. maurus* | 8,750008 | -67,541688 | TCWC 47883 |
| *E. maurus* | 8,708342 | -67,541688 | TCWC 47888 | *E. maurus* | 8,666675 | -78,166683 | KU 110272 |
| *E. maurus* | 8,666675 | -67,541688 | TCWC 47887 | *E. maurus* | 8,583342 | -67,583354 | TCWC 47886, 47259 |
| *E. maurus* | 8,541675 | -67,625021 | TCWC 53478 | *E. maurus* | 8,541675 | -66,041688 | IBSP 18408 |
| *E. maurus* | 8,416675 | -80,500016 | KU 110269 | *E. maurus* | 8,375009 | -80,166683 | KU 110271 |
| *E. maurus* | 8,333342 | -80,500016 | KU 110270 | *E. maurus* | 8,208342 | -80,958349 | TCWC 19251 |
| *E. maurus* | 8,166675 | -77,666684 | BNHM 83430, 83432 | *E. maurus* | 8,125009 | -77,70835 | MCZ R-177154, R-177155, R-177156  NMNH-USNM 140669 |
| *E. maurus* | 8,083342 | -81,000016 | BNHM 78740 | *E. maurus* | 7,833342 | -80,375016 | KU 107640 |
| *E. maurus* | 7,583342 | -80,125016 | KU 61349 |  |  |  |  |

* The latitude and longitude correspond to the center of the 5 × 5 km pixel where there is a presence site.

# Capture localities of specimens used in the phylogenetic analysis

BNHM: Berkeley Natural History Museums, Berkeley, USA

## **CAS: California Academy of Natural Sciences, California, USA**

## CMNH: Carnegie Museum of Natural History, Pittsburgh, USA

FML: Fundación Miguel Lillo, San Miguel de Tucumán, Argentina

## FPR: Fundación Puerto Rastrojo, Bogotá, Colombia

IBSP: Instituto Butantan, Sao Paulo, Brazil

KU: Natural History Museum, University of Kansas, Kansas, USA

LACM: Los Angeles County Museum of Natural History, Los Angeles, USA

MACN: Museo Argentino de Ciencias Naturales, Buenos Aires, Argentina

MCZ: Museum of Comparative Zoology, Harvard University, Cambridge, USA

MHN-UNICAMP: Museu de Historia Natural, Universidade Estadual de Campinas, Campinas, Brazil

## MZUEL: **Museu de Zoologia da Universidade Estadual de Londrina, Londrina, Brazil**

NMNH-USNM: National Museum of Natural History, Smithsonian Institution, Washington, DC, USA

## TCWC: **Texas Cooperative Wildlife Collection, Texas, USA**
